# Google Classroom as a Teaching Tool for Undergraduate Embryology

**DOI:** 10.7759/cureus.29701

**Published:** 2022-09-28

**Authors:** Sunit Jadhav, Oshin Behl, Ananya Khurjekar, Varun Pathak, Akatya Sinha, Arunprasad VK

**Affiliations:** 1 Anatomy, Symbiosis Medical College for Women, Pune, IND; 2 Otolaryngology, Regional Hospital Pardubice and University of Pardubice, Pardubice, CZE; 3 Medicine, B J Government Medical College, Pune, IND; 4 Medicine, Mahatma Gandhi Memorial Medical College, Mumbai, IND

**Keywords:** anatomy, embryology, medical education, learning management system, google classroom, e-learning

## Abstract

Context and aim

Modern teaching of medicine has evolved into a beyond-the-classroom experience. Learning management systems (LMSs) have made this possible because of easy accessibility and user-friendliness. The COVID-19 lockdown further accentuated the need for this mode of education delivery. General embryology (GE) is a subject under human anatomy that does not rely on “touch-and-feel” as much as other medical subjects. Assess Google Classroom (GC) as a teaching tool to deliver an online undergraduate-level general embryology (GE) course.

Settings and design

A cross-sectional study involving 211 undergraduate medical students across India.

Methods and material

A pre-and post-quiz model was adopted to evaluate the efficiency of a five-lecture course on GE. The course content was delivered via 20-minute YouTube video lectures, uploaded on GC. Lastly, student feedback regarding gadget preferences and the overall learning experience was collected. Statistical Analysis: The confidence interval was set at 95%, and a p-value </= 0.05 was considered statistically significant. Data were analyzed using SPSS software v.23.

Results

We observed a significant increase in mean quiz scores of all quizzes with increased perceived confidence in the subject, yet a high attrition rate (74.8 %). GC scored a 4.3/5 for user-friendliness, and 83.7% of students preferred cell phones for accessing GC.

Conclusion

GC, with its limitations, poses a significant challenge to teaching GE online. Software updates in the future might prove it to be a competent alternative to other LMSs in the market. GC falls short in terms of providing optimum levels of assessment and interaction for learning complex topics like embryology.

## Introduction

In today’s modern world, teaching and learning are no longer confined to the chalk-and-talk method. Nowadays, teachers are expected to provide a better learning environment and facilities to students inside and outside the classroom to encourage “beyond the classroom” learning [1]. This has become easier to achieve thanks to the implementation of technology in education. The internet has freed education from the shackles of space, distance, and time. E-Learning, delivered via learning management system (LMS) software, now holds the potential to transform “learning with effort” into “learning with fun” [2]. Distance education has recently acquired great popularity amongst educational institutions, and the incorporation of massive open online courses has revolutionized universities and the corporate education landscape [3]. In early 2020, the shutting down of educational institutions worldwide owing to the coronavirus pandemic further accelerated this rather aggressive shift toward online education. The medical teaching fraternity was no exception. Cross-sectional studies done in medical schools in China suggest low adaptability but a positive opinion of this change [4]. In a middle-income country like India, user compatibility and cost efficiency are the two most important factors to be considered while undertaking this sudden transition.

Google Classroom (GC), a virtual class launched by Google Apps for Education (GAFE) in 2014, is one such LMS that meets both criteria. Developed by Google, GC enables teachers and students to engage in beyond-the-classroom learning innovatively. It allows teachers to expand their classroom management and activities planning techniques and facilitates their classes, helps create and organize assignments quickly, and provides valuable feedback efficiently through an accessible mode of communication [5]. Furthermore, a recent 2014 update made GC a component of Google Apps Education, which encompasses a free suite of productivity tools, including Gmail, Google Drive, and Google Docs, thus enabling educators to use a virtual cloud for storing their data. The ease of access and simplicity of the user interface make GC a viable tool for educators who are relatively new to e-learning [6]. Online education continues to grow and plays an increasingly significant role not just in India but worldwide in higher education. This online environment has even encouraged feelings of social connectedness and group cohesion [7]. However, one must also bear the associated challenges of online learning in India [8]. A poor internet connection can lower students’ motivation and raise the chances of miscommunication between the student and teacher, especially if the instructions are not adequately clear [9]. Technological support from good quality computers and a stable internet connection is a prerequisite to minimizing these adversities. The teachers of human anatomy, a basic science taught to medical schools worldwide, confront an even more significant challenge because face-to-face interactions over cadaveric dissections have always played a pivotal role, particularly in teaching gross anatomy [10].

During the COVID-19 lockdown, this type of interactive teaching was not an option [11]. Developmental anatomy seemed like a more suitable alternative for continuously delivering anatomical knowledge to medical undergraduates at this time. Considering the reduced enthusiasm amongst medical students for the study of general embryology and its underestimated relevance in clinical practice, the authors thought that distance learning during the pandemic would provide a good opportunity to establish some basic embryological concepts [12].

A retrospective study comparing live versus online embryology teaching found that while the former allows better teacher-student interaction, the latter allows the student to pause and replay the lecture at a convenient time [13]. No studies have been conducted using GC as a teaching tool for embryology. In this study, the authors attempted to evaluate the efficacy of GC by delivering a short course on general embryology to undergraduate medical students in India.

## Materials and methods

This was a cross-sectional study that included a total of 211 undergraduate medical students from all over India. Consecutive sampling was done for four weeks before the commencement of the classroom lectures. Social media channels viz Whatsapp, Instagram, and e-mail were used to recruit 211 participants. Table 4.2, Serial Number, 1 of the National Ethical Guidelines for Biomedical and Health Research involving Human Participants of the Indian Council of Medical Research, New Delhi, studies comparing instructional techniques, curricula, or classroom management methods are exempted from Ethical Committee review. We used a pre- and post-quiz methodology to assess improvement in student performances in a five-lecture course on general embryology (GE) distributed over a ten-day preplanned schedule. A post-study Google Form questionnaire was then used to collect student feedback regarding the course. The course content was delivered via 20-minute YouTube video lectures with links posted in the GC. A classroom titled “GE” was created first, and then undergraduate medical students were approached via Whatsapp broadcasts and Instagram posts [14,15]. Interested students were enrolled via invitation links shared over e-mail.

The four types of questionnaires used in the study were (a) a single pre-study questionnaire (Q0), (b) five pre-lecture quizzes (PreQ1 to PreQ5), (c) five post-lecture quizzes (PostQ1 to PostQ5), and (d), a single post-study questionnaire (Q6). Subject experts validated the pre-and post-lecture quizzes. As soon as the students entered the class, they were asked to fill out Q0, the first-page containing information about the study, followed by consent to be given through a Yes/No question. Participation was voluntary, and data collection was done anonymously. An e-booklet explaining the predecided lecture schedule was then uploaded to the classroom. The lectures were uploaded one at a time, on alternate days starting from May 20, 2020, to May 28, 2020, all at 7 pm IST. The format of the uploading of the lecture series was as follows: (a) PreQ1 was uploaded 12 hours before the lecture, (b) the 20-minute lecture was uploaded at 7 pm IST, and (c) PostQ1 was uploaded 20-minutes after the lecture release and locked 15 minutes after uploading (Tables 1, 2).

**Table 1:** Lecture Schedule

Table 1: Lecture Schedule			
No.	Topic	Date	Time
1.	Cell Division I: Mitosis	20-05-20	7:00 pm
2.	Cell Division II: Meiosis	22-05-20	7:00 pm
3.	Gametogenesis I: Oogenesis	24-05-20	7:00 pm
4.	Gametogenesis II: Spermatogenesis	26-05-20	7:00 pm
5.	Embryogenesis - Week 01: Fertilisation & Implantation	28-05-20	7:00 pm

**Table 2:** Quiz Schedule

Table 2: Quiz Schedule					
No.	Quiz type	Topic	Quiz No.	Date	Time
1.	Pre- quiz	Cell Division I: Mitosis	PreQ_1_	20-05-20	7:00 am
2.	Post-quiz	Cell Division I: Mitosis	PostQ_1_	20-05-20	7:20 pm
3.	Pre- quiz	Cell Division II: Meiosis	PreQ_2_	22-05-20	7:00 am
4.	Post-quiz	Cell Division II: Meiosis	PostQ_2_	22-05-20	7:20 pm
5.	Pre quiz	Gametogenesis I: Oogenesis	PreQ_3_	24-05-20	7:00 am
6.	Post-quiz	Gametogenesis I: Oogenesis	PostQ_3_	24-05-20	7:20 pm
7.	Pre- quiz	Gameto- genesis II: Spermatogenesis	PreQ_4_	26-05-20	7:00 am
8.	Post-quiz	Gameto- genesis II: Spermatogenesis	PostQ_4_	26-05-20	7:20 pm
9.	Pre- quiz	Embryo- genesis - Week 01: Fertilisation & Implantation	PreQ_5_	28-05-20	7:00 am

Q0 collected student information about their prior experience with online medical education in general and with GC in particular, electronic device preferences for accessing online lectures, and about their confidence in the topics of GE to be covered, as well as overall confidence in the subject. All quizzes consisted of 10 single-response multiple choice questions (MCQs). For every lecture, the pre-and post-quizzes had identical questions, and the difference in the scores was used to assess the student’s progress on that topic. Although the pre-quiz could be filled out anytime within the 12-hour window preceding the lecture release, the post-quiz was to be filled out immediately after the lecture. To ensure this adherence to timing, it was locked 15 minutes after its upload. At the end of the five lectures, on the 10th day, Q6 was circulated among the participants via e-mail to gather feedback regarding their confidence levels in the topics taught, overall confidence in the subject, user-friendliness of GC in online education, reasons for discontinuity for those who dropped out of the study midway, and suggestions to improve this situation.

The confidence interval was set at 95%. A p-value less than or equal to 0.05 was considered statistically significant. Data were analyzed using SPSS software v.23 (IBM Statistics, Chicago, USA) and Microsoft Office 2007.

## Results

**Figure 1:**
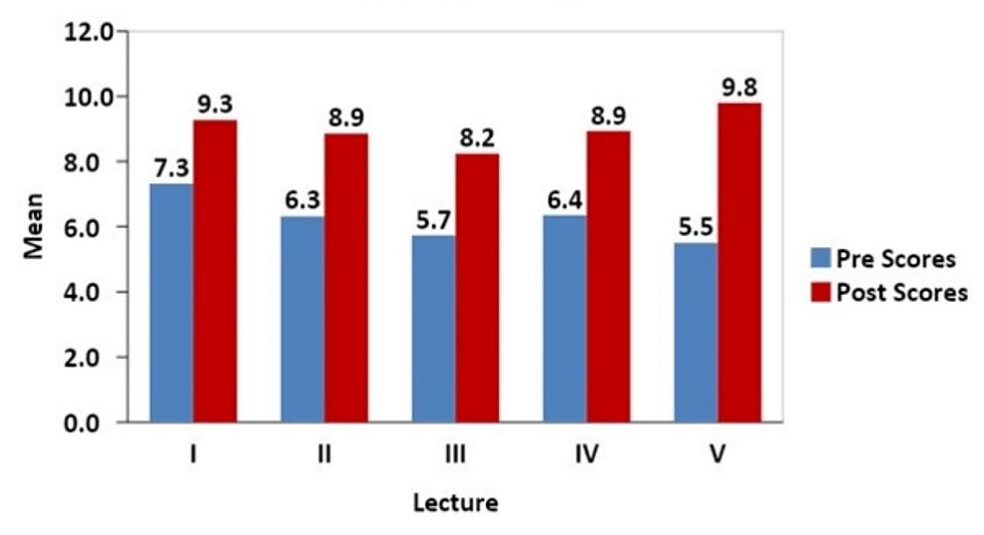
Comparison of Scores between Pre and Post Quiz

We observed a significant increase in both the mean quiz scores and the topic- and subject-related confidence levels. A high attrition rate was also observed amongst the participants. The mean scores rose in all five quizzes (p < 0.001), with the maximum rise in the last lecture (Embryogenesis- 5.5 to 9.8; Fig 1).

**Figure 2:**
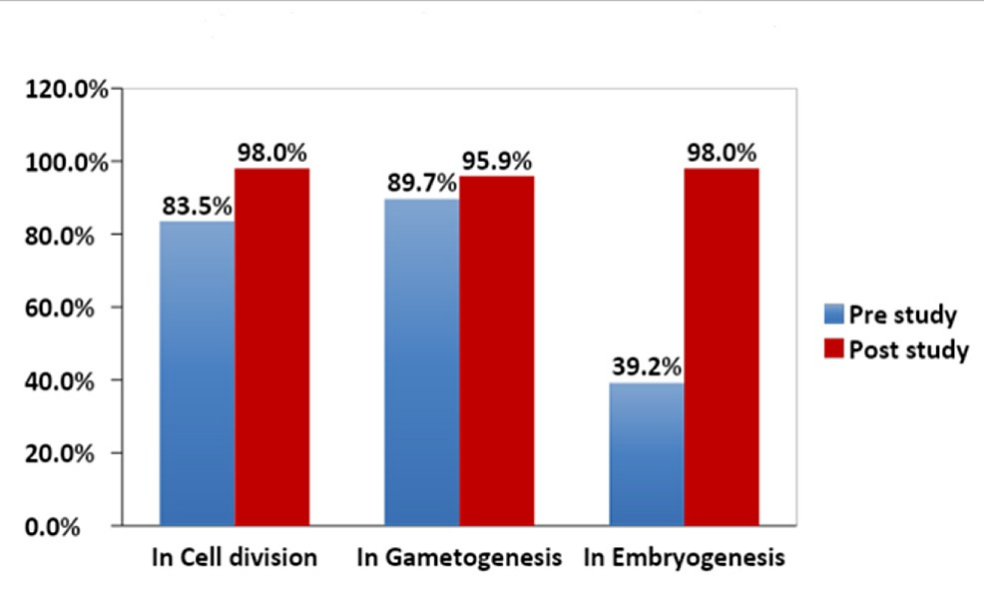
Overall Confidence of Subtopics in Pre and Post Lectures

An increase in student confidence was seen in the subject overall, as well as in the individual topics that the lectures covered. The confidence amongst the students in the topics of cell division (p = 0.01) and embryogenesis (p < 0.001) increased significantly after the study (p < 0.001), with the maximum growth being in embryogenesis, which shot up steeply to 98% from 39.2% (Fig 2).

**Figure 3:**
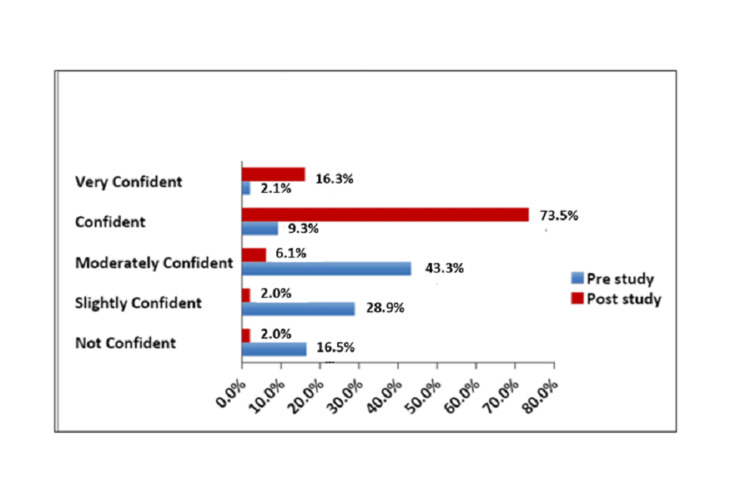
Overall level of Confidence in General Embryology in Pre and Post Lectures

An explanation for this spike could be that out of all the topics, and embryogenesis was the only one not touched upon in the Higher Secondary Certificate (HSC) biology curriculum. The overall confidence in general embryology also showed uniform growth (Fig 3).

**Figure 4:**
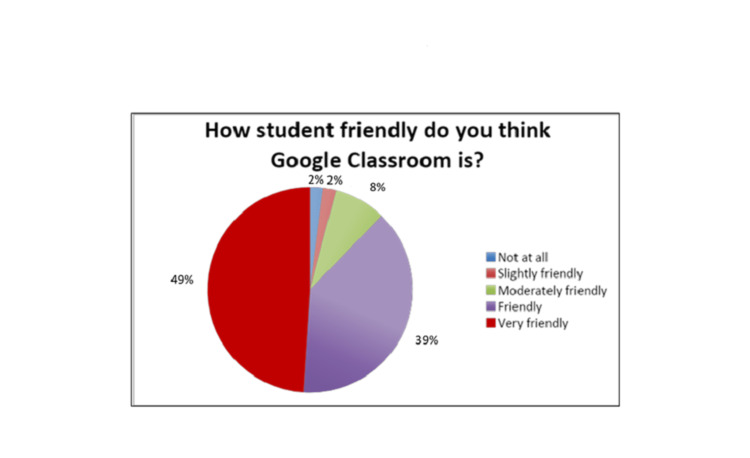
User Friendliness of Google Classroom

While only 9.3 % of students were confident about the subject pre-study, 73.5 % felt confident about it after attending the lectures. Students marked GC at 4.3 (out of 5) for its user-friendliness; 49% of students found it very student-friendly (Fig 4).

**Figure 5:**
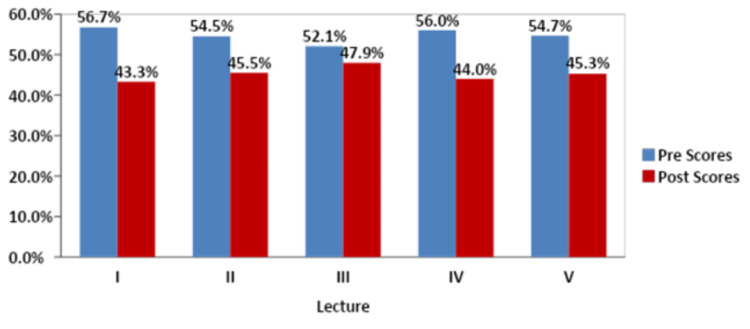
Distribution of participants between Pre and Post Quiz

An important observation was the significantly high attrition rate. Out of the 211 students that filled the pre-questionnaire Q1, only 127 (56.7%) attempted the first pre-quiz (q1), and only 97 students (43.3%) attempted the first post-quiz (pq1; Fig 5).

**Figure 6:**
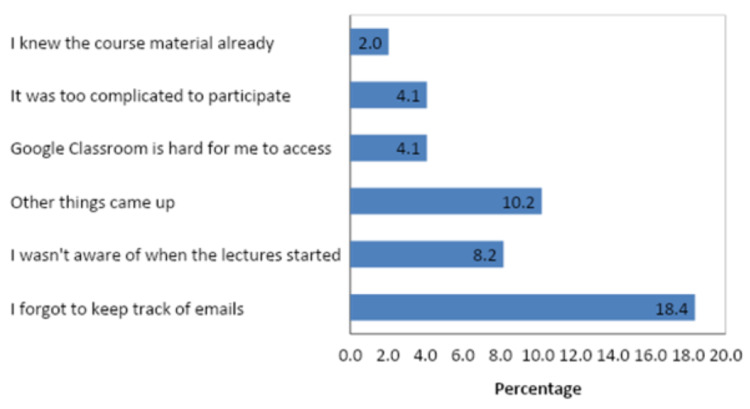
Reason for Non-Participation (Multiple Possible)

These numbers kept declining with every lecture, and the number of students who followed through with the study and filled out all quizzes up to pq5 was 53. A total of 49 students filled out the post-study questionnaire (Q2): only (23.2%). When asked about the discontinuity, 28.6% of students stated that it was due to other academic or nonacademic engagements (Fig 6).

## Discussion

The observations suggest that although GC is an excellent tool for eliminating the factor of physical distance in student learning, ensuring constant engagement that imitates a live classroom setting poses a significant challenge. The students who followed through with the entire lecture series reaped the benefits of increased scores and raised confidence. However, that alone does not indicate GC’s efficiency as a teaching tool, especially for teaching medical subjects that require aptitude and consistency. From the collected feedback, we deduced the following factors that led to the inability of most students to follow through with the lectures:

Inflexible lecture and quiz schedule

The sudden transition from live to online education during the first wave of the coronavirus outbreak was challenging for the students [16-19]. The inability to access the lectures and quizzes at a convenient time was a major reason for the lack of compliance. Although GC allows the freedom of accessing a posted YouTube link at any time, ensuring that the post quiz was filled out immediately after viewing the lecture was necessary. Multiple lecture views before attempting the post-quiz would have skewed the data. A variety of open-access LMSs now offer a quiz feature. The incorporation of such features could increase the efficiency of GC as a teaching tool, especially while focusing on the assessment part of education [20-22].

Delayed display of quiz scores

Many students dropped out because they had to wait until the end of the study to view all quiz scores, and this time gap demotivated them from continuing. In our scientific opinion, an increased or decreased confidence level perceived by the student after the release of the scores midway through would have affected their performance, thus creating a bias. The only viable option in such a case was to shorten the 48-hour gap between lectures. However, considering the academic burden of their college schedules, this increased the chances of their non-compliance.

Limited teacher-student interaction

Many students expressed dissatisfaction with the paucity of teacher-student interaction throughout the course, which was communicated directly to the lecturer. They favored group video call interactions over e-mail communications. Similar studies have established a link between video conferencing and better learning outcomes in the current online education situation [23,24]. Although an instructional video was created and posted in the classroom, the lack of a live question-answer session, where the students would get to virtually meet and bond with the teacher, diffused their interest. However, GC has the advantages of being free and user-friendly, the lack of inbuilt plug-ins, such as time-based quizzes with automatic grading, and direct private messaging between the teacher and the student. A conference call button for online classroom interactions makes it a teaching tool with much room for improvement, especially for medical training, which involves much teacher-learner engagement.

Since the advent of the pandemic, the need for higher education organizations to adopt e-learning courses as part of their curriculum has increased dramatically, thus initiating the discussion of which LMS would be the most suitable for the task. In a qualitative study in January 2010, Cigdem et al. [25] compared 13 open-source LMSs under multiple points such as user authenticity, file support, compatibility, online assessments, and video conference support. They concluded that Moodle, with its modular design and greater attention to the user interface, was the most suitable LMS for higher educational organizations. In 2019, Dash used GC to conduct a series of 13 lectures on medical biochemistry and collected feedback from 41 medical students [26]. However, the methodology did not involve pre- and post-quizzes; hence the feature of planning a specific time window for post-quiz submission was not required. A better learner evaluation requires an LMS that offers better assessment features. The increasing number of open-source LMSs currently behoves modern educators to explore more possibilities and expand their horizons in terms of e-learning-related research.

Limitations

The primary limitation of our study was the high attrition rate, prompting the question of whether there was a loss of follow-up bias. Consequently, attrition led to a decreased sample size, which would naturally impact the power of the study. Additionally, the study could have been more comprehensive, considering that it evaluated the efficacy of a widely used online teaching tool. Thus, we propose that subsequent studies can overcome this limitation by implementing a broader framework of lectures and a longer study duration.

## Conclusions

With its current limited features, it is debatable whether GC holds the potential to be an institute-level LMS. Software updates in the future might prove it to be a competent alternative to other LMSs in the market. However, given the immediate need to switch to a more engaging and advanced form of online education, especially with the unpredictable intermittent shutting down of medical colleges to prevent COVID-19 transmission, GC falls short in terms of providing optimum levels of assessment and interaction for learning complex subjects such as medical embryology.

## References

[1] Patrick S, Sturgis C (2015). Maximizing Competency Education and Blended Learning: Insights from Experts. INACOL, The International Association for K-12 Online Learning. https://eric.ed.gov/?id=ED557755.

[2] Rusman MP (2016). The development of an e-learning-based learning service for MKDP curriculum and learning at the Indonesia University of Education. J Educ Pract.

[3] Kaplan AM, Haenlein M (2016). Higher education and the digital revolution: about MOOCs, SPOCs, social media, and the cookie monster. Bus Horiz.

[4] Li W, Gillies R, He M, Wu C, Liu S, Gong Z, Sun H (2021). Barriers and facilitators to online medical and nursing education during the COVID-19 pandemic: perspectives from international students from low- and middle-income countries and their teaching staff. Hum Resour Health.

[5] Sophia MM, Sasirekha M, Ebenezer JL, Vaijayanthimala P (2020). Perception of undergraduate medical and dental students towards learning anatomy in google classroom. Indian J Public Health Res Dev.

[6] Shaharanee INM, Jamil J, Rodzi SSM (2016). The application of Google Classroom as a tool for teaching and learning. J Telecommun Electron Comput Eng.

[7] Zydney JM, Seo KKJ (2012). Creating a community of inquiry in online environments: an exploratory study on the effect of a protocol on interactions within asynchronous discussions. Comput Educ.

[8] Nimavat N, Singh S, Fichadiya N (2021). Online medical education in India - different challenges and probable solutions in the age of COVID-19. Adv Med Educ Pract.

[9] Dhingra S, Pasricha N, Sthapak E, Bhatnagar R (2021). Assessing the role of internal motivation and extrinsic factors on online undergraduate medical teaching in a resource-poor setting during COVID-19 pandemic in North India: an observational study. Adv Med Educ Pract.

[10] Ghosh SK (2017). Cadaveric dissection as an educational tool for anatomical sciences in the 21st century. Anat Sci Educ.

[11] Singal A, Bansal A, Chaudhary P (2020). Cadaverless anatomy: darkness in the times of pandemic Covid-19. Morphologie.

[12] Hamilton J, Carachi R (2014). Clinical embryology: is there still a place in medical schools today?. Scott Med J.

[13] Beale EG, Tarwater PM, Lee VH (2014). A retrospective look at replacing face-to-face embryology instruction with online lectures in a human anatomy course. Anat Sci Educ.

[14] Alsleem H, Alsleem RH, Adam M, Ibrahim K, Saad M, Almohiy NH, Hadi NH (2019). The role of Whatsapp in scientific education at the college of applied medical sciences at Imam Abdulrahman bin Faisal University. King Khalid University Journal of Health Sciences.

[15] Carpenter JP, Morrison SA, Craft M, Lee M (2020). How and why are educators using Instagram?. Teach Teach Educ.

[16] Dedeilia A, Sotiropoulos MG, Hanrahan JG, Janga D, Dedeilias P, Sideris M (2020). Medical and surgical education challenges and innovations in the COVID-19 era: a systematic review. In Vivo.

[17] Goswami S (2020). Online education in Corona outbreak: a challenge, boon or curse in India.

[18] Pacheco AQ (2009). Issues for effective distance learning: a challenge in online education. Revista de Lenguas Modernas.

[19] Edelhauser E, Lupu-Dima L (2021). One year of online education in Covid-19 age, a challenge for the Romanian education system. Int J Environ Res Public Health.

[20] Dougiamas M (2004). Moodle: a virtual learning environment for the rest of us. The Electronic Journal for English as a Second Language.

[21] Gamage SH, Ayres JR, Behrend MB, Smith EJ (2019). Optimising Moodle quizzes for online assessments. Int J STEM Educ.

[22] Memon AR, Rathore FA (2018). Moodle and online learning in Pakistani medical universities: an opportunity worth exploring in higher education and research. J Pak Med Assoc.

[23] Lamba P (2011). Teleconferencing in medical education: a useful tool. Australas Med J.

[24] Allen M, Sargeant J, Mann K, Fleming M, Premi J (2003). Videoconferencing for practice-based small-group continuing medical education: feasibility, acceptability, effectiveness, and cost. J Contin Educ Health Prof.

[25] Aydin CC, Tirkes G (2010). Open source learning management systems in distance learning. Turkish J Educational tech.

[26] Dash S (2019). Google classroom as a learning management system to teach biochemistry in a medical school. Biochem Mol Biol Educ.

